# Molecular identification of *carnivore chaphamaparvovirus 2* (feline chaphamaparvovirus) in cats with diarrhea from China

**DOI:** 10.3389/fvets.2023.1252628

**Published:** 2023-10-03

**Authors:** Hao Cui, Zhibin Zhang, Xin Xu, Kejing Zuo, Jun Ji, Ge Guo, Yunchao Kan, Lunguang Yao, Qingmei Xie, Yingzuo Bi

**Affiliations:** ^1^Henan Provincial Engineering Laboratory of Insects Bio-reactor, Nanyang Normal University, Nanyang, China; ^2^Laboratory Animal Center, Chifeng Municipal Hospital, Chifeng, China; ^3^Guangzhou Zoo and Guangzhou Wildlife Research Center, Guangzhou, China; ^4^South China Collaborative Innovation Center for Poultry Disease Control and Product Safety, South China Agricultural University, Guangzhou, China

**Keywords:** pet cats, feline chaphamaparvovirus, rectal swabs, phylogenetic tree, recombination analysis

## Abstract

*Chaphamaparvovirus carnivoran2* (feline chaphamaparvovirus, FeChPV) is a novel feline parvovirus originally detected in Canadian cats in 2019, and it has also been identified in domestic cats in other nations. To evaluate the prevalence and genetic diversity of FeChPV in China, rectal swabs of pet cats from Henan, Guangdong, Anhui, Zhejiang, and Inner Mongolia provinces were collected. Of the 230 samples subjected to nested polymerase chain reaction, 6 (2.6%) tested positive for FeChPV. Although all positive samples were from cats with diarrhea, statistical analyses revealed no correlation between the presence of the virus and clinical symptoms (*p* > 0.05). Phylogenetic trees of nonstructural protein 1 (NS1) and capsid protein (VP1) demonstrated that these six new strains formed a major branch with other reference FeChPV strains and considerably differed from *Chaphamaparvoviru carnivoran*1. Moreover, recombination analysis revealed that the FeChPV strain CHN20201025, previously detected in a dog, was a recombinant and strains CHN200228 and CHN180917, identified in this study, were the closest relatives to the parental strains. The findings of this study and a previous study wherein FeChPV was detected in dogs suggest that FeChPV can propagate between species. Additionally, these findings indicate that the genetic diversity of FeChPV can provide an insight into the epidemiological status of FeChPV in China.

## 1. Introduction

In the family *Parvoviridae*, parvoviruses are small, nonenveloped, icosahedral-shaped viruses with single-stranded DNA genomes that can range from 3.9 kb to 6.3 kb in length (1). The *Hamaparvovirinae* subfamily comprises *Penstylhamaparvovirus*, *Brevihamaparvovirus*, *Hepanhamaparvovirus*, *Chaphamaparvovirus*, and *Ichthamaparvovirus* (2, 3).

Owing to the improvements in detection and sequencing technologies, chaphamaparvoviruses were identified in several hosts via high-throughput sequencing in the last few years. Their hosts are Tasmanian devils (4), bats (5, 6), mice (7, 8), pigs (9), turkeys (10), and chickens (11). Subsequently, chaphamaparvoviruses detected in dogs and cats were recognized as *Chaphamaparvovirus Carnivoran1* (CachaV, termed as cachavirus) and *Chaphamaparvovirus Carnivoran2* (feline ChPV, termed as fechavirus), respectively (12, 13). Fechavirus (FeChPV) was initially identified in the feces of Canadian cats that had suffered from an outbreak of acute gastroenteritis in 2019 (13); however, FeChPV DNA was subsequently detected in cats with or without gastroenteritis signs and those with or without upper respiratory tract disease (URTD); statistics suggested the association of this virus with acute gastroenteritis (14). Notably, FeChPV was also reported in a Chinese cat shelter, and the prevalence rate of FeChPV among cats exhibiting signs of URTD is as high as 81.08%. This report also speculated that FeChPV can replicate in immunological organs of cats and lead to URTD, encephalitis, and lymphadenitis (15). Despite these data, the potential health impacts of FeChPV on cats and its potential as an intestinal pathogen warrants further investigation.

In this study, samples obtained from healthy domestic cats and cats with diarrhea were analyzed and FeChPV was studied to improve the understanding of its epidemiology and evolution in China.

## 2. Materials and methods

### 2.1. Sample collection

Rectal swabs from 230 cats (45 healthy cats and 185 cats with diarrhea) were obtained from pet hospitals in Guangdong, Henan, Anhui, and Zhejiang provinces and the Inner Mongolia Autonomous Region from June 2018 to March 2021. This study adhered to the ethical policies and was approved by the Committee on the Ethics of Animal Experiments of South China Agricultural University (SYXK 2019-0136).

### 2.2. Nucleic acid extraction and FeChPV detection

For nucleic acid extraction, sodium phosphate buffered saline was added to swabs collected from cats. They were then vortexed and centrifuged at 4°C. Viral DNA/RNA was extracted from these samples using the EasyPure Viral DNA/RNA Kit (TransGen Biotechnology, Beijing, China). In this investigation, diagnostic primers for the nonstructural protein 1 (NS1) protein-coding gene (sited 2138–2448 according to the IDEXX-1 strain; accession no. MN396757) were used to determine the presence of FeChPV DNA through a previously reported hemi-nested polymerase chain reaction (PCR) method. The primers used for the analysis are listed in Supplementary Table S1 (14). First-round PCR primers (FeChPVF1 and FeChPVR1) amplified a 332 bp region, and second-round primers (FeChPVF2 and FeChPVR2) amplified a 311 bp region. The reaction conditions were as follows: 95°C for 5 min, 30 cycles at 95°C for 30 s, 58°C for 30 s, 72°C for 30 s, and a final extension at 72°C for 10 min.

### 2.3. Diagnostic PCR for other feline viruses related to diarrhea

The samples were examined for feline gastroenteritis–associated viruses other than FeChPV, such as cachavirus (CachaV) (12), feline astrovirus (FeAstV) (16), feline bocavirus (FBoV) (16), feline kobuvirus (FeKoV) (17), and feline parvovirus (FPV) (18), using reported primer pairs that exhibit extensive reactivity with various viruses. The primer pairs used are listed in Supplementary Table S1. Moreover, the UpSet diagram produced by TBtools and Wayne diagram^1^ were jointly used to indicate the viral coinfection status (19, 20).

### 2.4. Complete genome sequencing

Based on the genome sequences of the FeChPV strain IDEXX-1, eight primer pairs amplifying overlapping fragments were designed and synthesized (Supplementary Table S1) to amplify the whole genome of FeChPV. For PCR, a 20-μL reaction mixture was prepared, which included a template DNA (>100 ng/L), 6 pmol of upstream and downstream primers, primer STAR HS DNA polymerase, and supporting reaction buffer (TaKaRa). For sequence amplification, the following cycle conditions were employed: predenaturation at 95°C for 5 min, followed by 35 cycles of 95°C for 30 s, 55°C for 30 s, and 72°C for 1 min, and the final extension step at 72°C for 10 min. The harvested amplicons were inserted into pMD18-T cloning vector (TaKaRa) and sequenced by Hongxun Company (Jiangsu, China).

### 2.5. Sequence identification and phylogeny

The complete nucleotide sequence of the genome and the amino acid (aa) sequence of NS1 of the obtained and reference parvovirus strains were aligned via the Muscle algorithm in MEGA software (21). Phylogenetic trees were constructed using the aa sequences of NS1 and VP1 via the maximum likelihood method in MEGA software with the JTT + G + I model and 1,000 bootstrap replicates. Additionally, ChiPlot^2^ was used to construct and display the sequence alignment heatmap (22).

### 2.6. Recombination prediction

The prediction of recombination events in the obtained strains compared with other ChPVs was performed using RDP4.36 with default parameters via RDP, GENECONV, MAXCHI, and BOOTSCAN. The final results were confirmed using SimPlot 3.5.1 (23, 24).

### 2.7. Analysis of aa mutations in NS1 and VP1

To comprehensively understand the impact of mutation sites on the tertiary structure, CHN190305 and CHN191011 harboring representative variant sites were selected to construct structural models of NS1 and VP1, respectively. Moreover, tertiary structural models of NS1 and VP1 were constructed for the reference strain IDEXX-1. WeMol^3^ and SWISS-MODEL^4^ were used to model the altered aa sequences of the obtained strains. Furthermore, PyMOL was used to gather and preserve the modeled Protein Data Bank files.

### 2.8. Viral isolation

Supernatants from FeChPV-positive samples were cocultured with CRFK and MDCK cell lines for 4 days. The cultures were split every 4 days for five passages. PCR assay was performed using the method described above to detect FeChPVs.

### 2.9. Statistical analysis

The prevalence of FeChPV was compared between healthy and diarrheal cats using Fisher’s exact test. GraphPad Prism 8.0 (San Diego, California, United States) was used for all statistical analyses. Statistics were deemed significant at *p* < 0.05.

## 3. Results

### 3.1. Clinical history and virus screening

**Figure 1:**
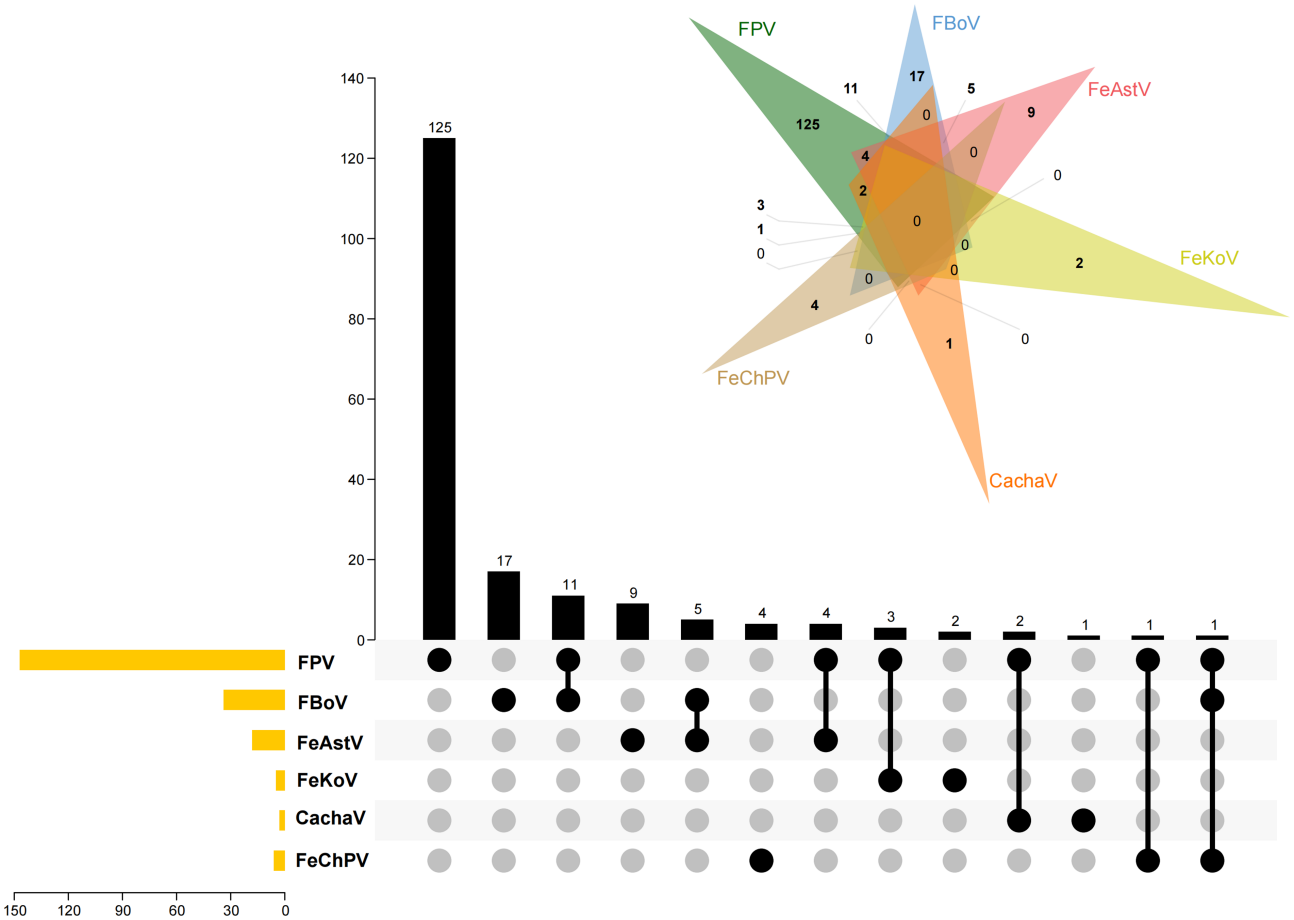
The status of infection by FeChPV, FPV, FBoV, FeAstV, FeKoV, and cachavirus. The Venn diagram and the UpSet plot depict the infection and coinfection by the viruses. In the UpSet plot, the sample numbers in each group are shown in the bar graph above. The positive sample numbers of each pathogen are depicted in the bar chart in the lower left corner. The infections in each group are shown using the dotted line in the bottom right corner.

Of the 185 collected samples, the 6 samples from cats with diarrhea tested positive for FeChPV. Samples from healthy cats did not test positive for FeChPV. In addition, 147 (79.4%), 34 (18.5%), 18 (9.7%), 5 (2.7%), and 3 (1.6%) samples tested positive for FPV, FBoV, FeAstV, FeKoV, and CachaV in cats with diarrhea, respectively (Figure 1). All FeChPV-positive samples (6 of 185, 3.2%) were from cats with symptoms of diarrhea. However, statistical analysis did not suggest an association between FeChPV infection and clinical symptoms (*p* > 0.05).

Additionally, cultivated cells did not exhibit cytopathic effect until the fifth passage, and FeChPV DNA could not be identified using PCR.

### 3.2. Identity analysis

The near full-length genome sequences (4,092 nucleotides) of six strains identified in this study were deposited in GenBank (accession nos. OQ694028, OQ694029, OQ694030, OQ694031, OQ694032, and OQ694033). The comparison of whole-genome sequences of the six strains and the reference FeChPV strains deposited in the National Center for Biotechnology Information database demonstrated that the FeChPV strains identified in this study contained two main open reading frames encoding a 658-aa NS1 and 508-aa VP1.

Sequence analyses revealed that the six strains possessed a complete genome nucleotide identity of 98.2%–99.1%. In addition, they exhibited 83.2%–98.9% and 61.2%–69.1% genome identity with other relevant FeChPV and CachaV strains, respectively. Moreover, the genome identities of the other viruses belonging to the genus *Chaphamaparvovirus* varied from 32.2% to 54.3% (Supplementary Figure S1). According to aa identity analysis of NS1 and VP1, the six FeChPVs shared overall aa identities of 96.6%–98.1% for NS1 and 97.8%–99.1% for VP1. The six FeChPVs had aa identities of 97.1%–99.1% for NS1 and 97.4%–99.2% for VP1 with FeChPV; 63.9%–65.3% for NS1 and 63.5%–65.2% for VP1 with CachaV strains, and 30.5%–47.5% for NS1 and 6.2%–48.6% for VP1 with other representative strains.

### 3.3. Phylogenetic analysis

**Figure 2:**
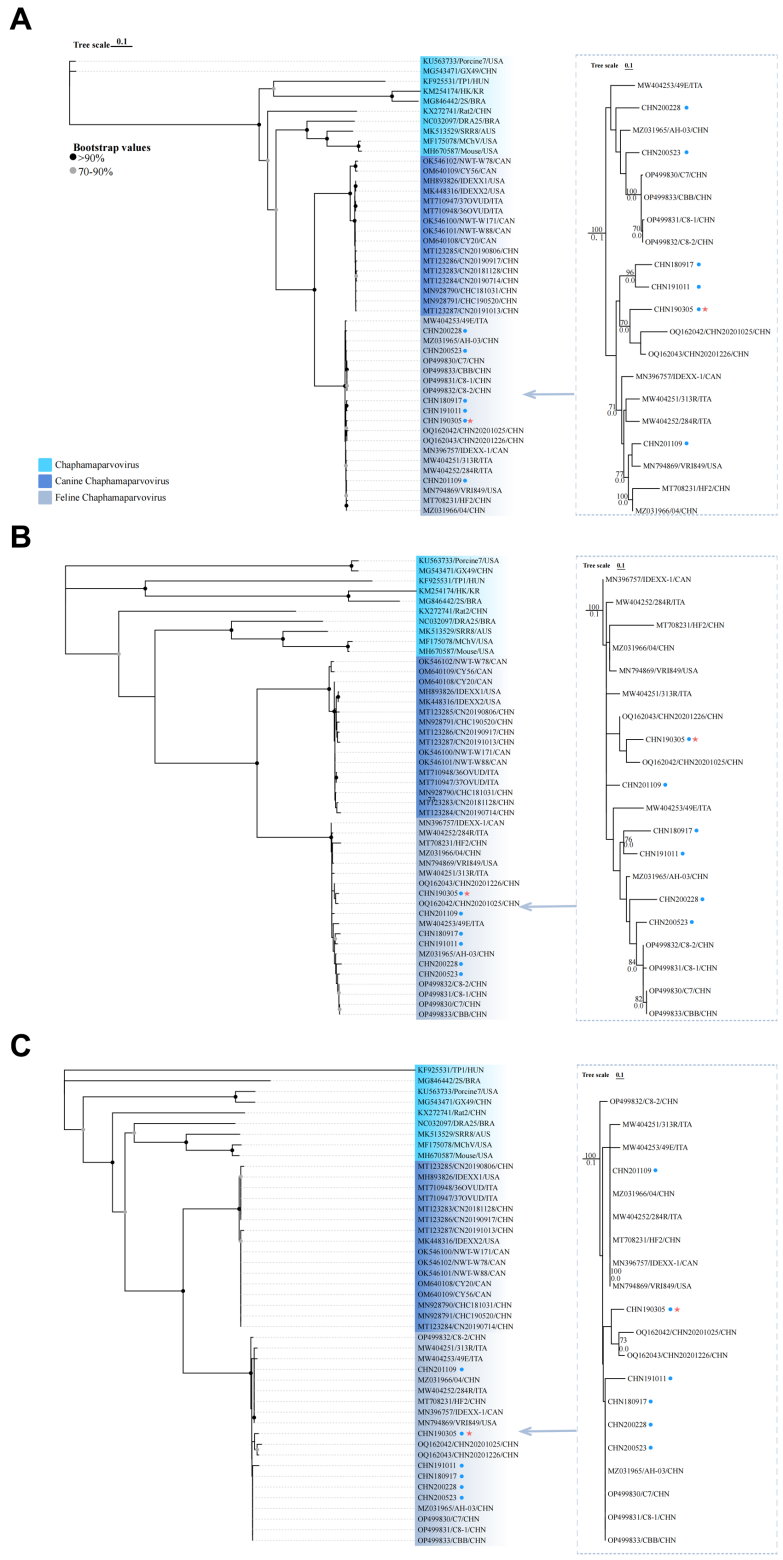
Evolutionary trees of the complete genome **(A)** and NS1 **(B)** and VP1 **(C)** amino acid sequences of FeChPV and other reference strains retrieved from GenBank. The separated trees on the right were for FeChPVs only. Bootstrap values below 70 have been hidden. The six obtained strains in this study are marked with a blue solid circle. The CHN190305 strain closely related to FeChPV previously found in dog are marked with red star.

Complete genome phylogenetic trees of the six obtained strains and 38 reference strains (FeChPV, CachaV, and other chaphamaparvovirus) were constructed to assess the genetic association (Figure 2A). The trees of FeChPV indicated that the two obtained strains CHN200228 and CHN200523 were closely related to reference FeChPVs, which were primarily identified from China with identities of 82.96%–98.66%. The remaining four FeChPVs were clustered into another sub-branch with CHN20201025 and CHN20201226 (detected in dogs, shared identities of 98.12%–99.02%) and reference strains detected in other countries. For the aa phylogenetic tree of NS1, the six obtained strains and other FeChPV strains were grouped into one major branch and were distantly related to cachavirus strains and reference ChPV strains (Figure 2B). The phylogenetic tree constructed using the aa sequences of VP1 showed that all the six obtained strains were clustered with two FeChPV strains from dogs and other Chinese FeChPV strains (except C8-2) previously reported (Figure 2C) (15, 25).

### 3.4. Recombination prediction

**Figure 3:**
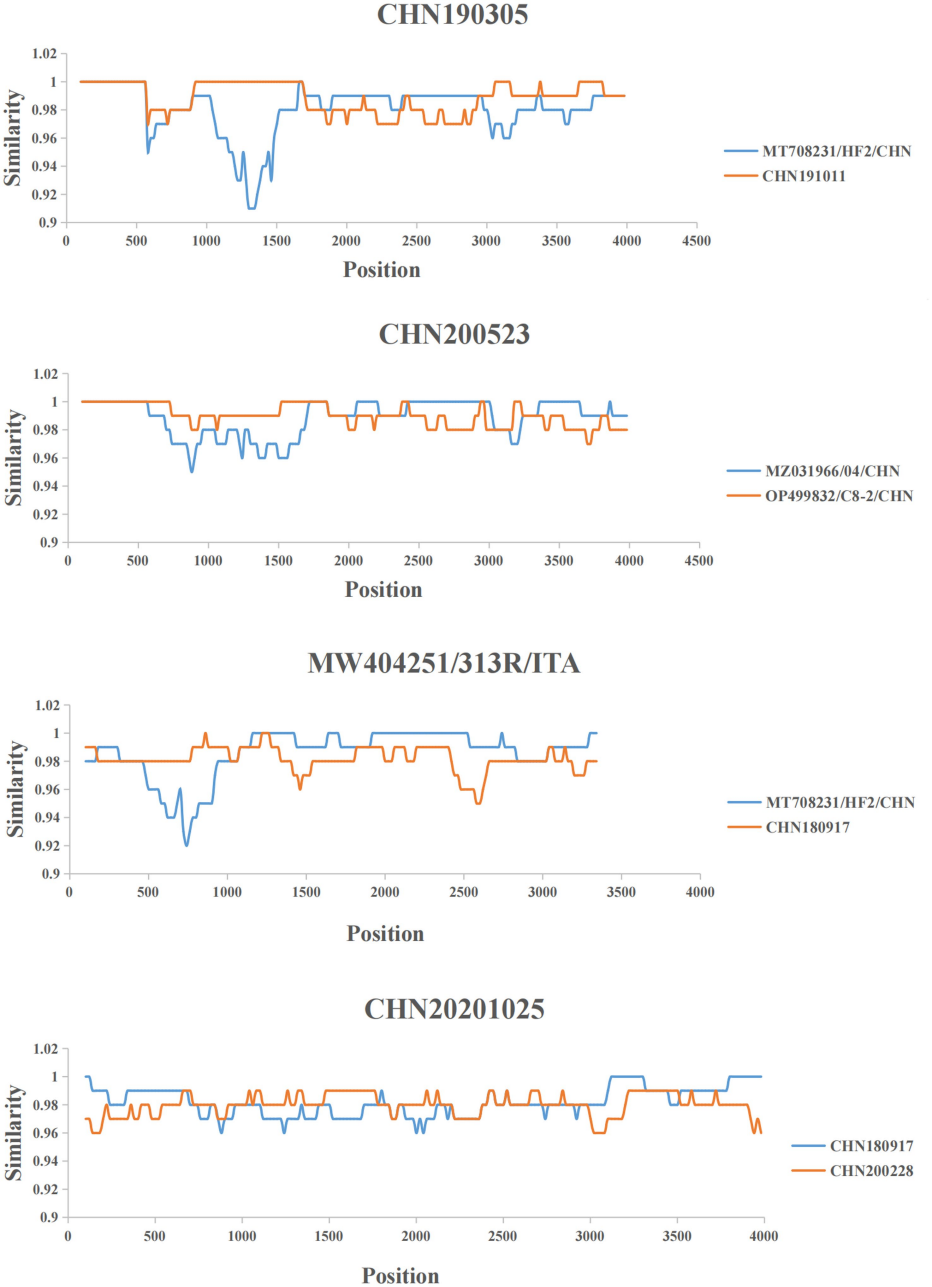
Representative recombined strains and predicted recombination events. Nucleotide identity plot comparing the nearly complete genome of strain CHN190305, CHN200523, MW404251/313R/ITA and CHN20201025, was performed by using Simplot according to the four representative recombination events. The recombination breakpoint is indicated by the intersection point of the two lines.

RDP4 and SimPlot were used to predict the recombination events in FeChPV strains (Supplementary Table S2). The recombination analysis predicted four recombinant events, indicating the two obtained strains CHN200228 and CHN180917 as minor and major recombinant parents, respectively, for CHN20201025 detected in dogs (Figure 3).

### 3.5. Mutation sites and structural modeling

**Figure 4:**
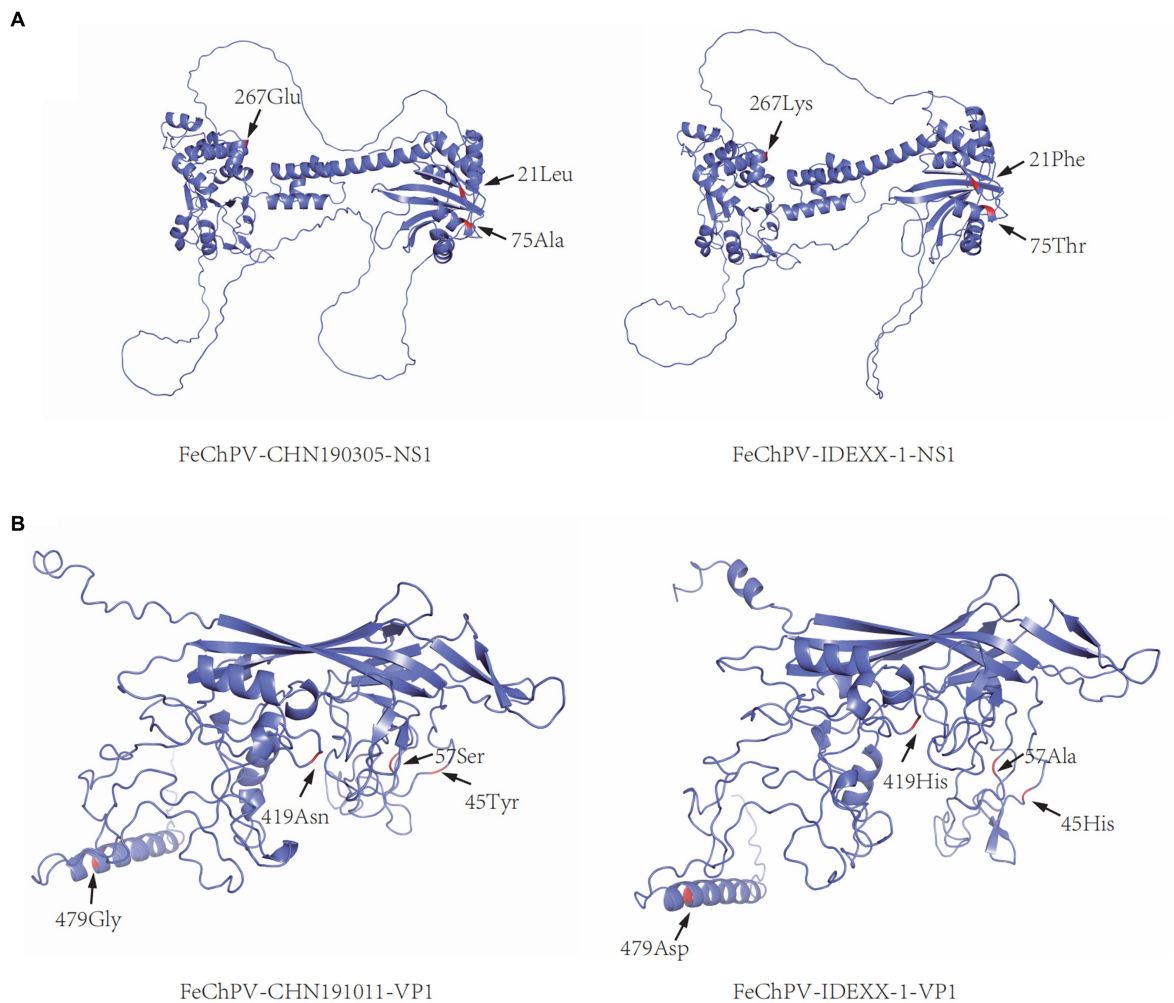
Tertiary structural change in the coding protein of FeChPV. **(A)** Predicted structural model of NS1 (FeChPV-CHN190315: representative strain identified in this study; FeChPV-IDEXX-1: representative reference strain). **(B)** Predicted structural model of VP1 (FeChPV-CHN191011: representative strain identified in this study; FeChPV-IDEXX-1: representative reference strain). “←” and letters marked with red indicate aa mutations.

The comparison of aa sequences of NS1 and VP1 in the six obtained FeChPVs and reference FeChPV strains revealed some unique variants. There were three unique sites in NS1, namely, Phe21Leu, Lys267Arg, and Lys267Glu/Arg, which were identified only in the obtained strains. Thr75Ala was found only in the NS1 protein of the obtained strains and few Chinese FeChPVs. The VP1 protein of the obtained FeChPVs demonstrated alterations at His419Thr and Asp479Gly. His45Tyr, Ala57Ser, and His419Asn were only harbored in the VP1 protein of the obtained strains and few Chinese FeChPVs. Supplementary Tables S3, S4 list the additional variant sites in NS1 and VP1, respectively. The variation in the tertiary structure of NS1 and VP1 of the obtained strains is illustrated in Figure 4.

Based on a previous study (26), it was inferred that the NS1 protein of the six obtained Chinese strains possessed two conserved endonuclease (replication initiator) motifs: 95-FHIHVIMAL-102 and 149-SLIAYMCK-156. Furthermore, motif related to the helicase domains 311-GCSNTGKS-318, 349-IGVWEE-354, 366-KQIFEGMECSIPVK-379, and 391-IIMTTN-396 was conserved in NS1 of all obtained strains except the CHN180917 strain, and it harbored the mutation of Met372Thr that did not lead to tertiary-structure change (27). The VP1 protein of other members of the subfamily *Hamaparvovirinae* did not possess the motifs of phospholipase A2 (PLA2) (28). Meanwhile, an 18 aa-elongation located at the N-terminus of the VP1 protein was present in all six strains obtained in this study and FeChPVs previously detected in Canada and Italy (14). A relatively long capsid protein has also been observed in other members of the genus *Chaphamaparvovirus*, which has been hypothesized to be related to low expression in host (29).

## 4. Discussion

The number of studies focusing on infectious diseases of pets is rapidly increasing. With the increase in the number of pets, pet-related intestinal diseases, such as FeAstV infections, have received considerable attention (30). Owing to the increasing use of metagenomic sequencing methods, several new enterovirus candidates have been identified (31, 32). The metagenomic sequencing methods, requires no prior knowledge of the microbial sequences present in the sample, and permits the evaluation of complex microbial communities without the need for the isolation and cultivation of individual microbial species (31, 33–35). The new FeChPV is suspected to cause vomiting and diarrhea in cats (13). However, the possible influence of this virus on cat health is yet to be elucidated.

A previous study on Chinese FeChPV detected in cats exhibiting URTD-related symptoms suggested feline calicivirus (FCV) and feline herpesvirus-1 as main viral pathogens and indicated that the URTD-related pathogenic ability of FeChPV is not explicit (15). Similar to the results reports in a study on FeChPV isolated from healthy cats in Turkey in 2022, statistical analysis in this study suggested the lack of correlation between FeChPV infection and clinical symptoms (36). We only detected ChPVs in swab samples from animals who had diarrhea. Two cats had concomitant with FPV and FeChPV infections, and one cat was also infected with FBoV. These findings indicate the need of exploring the pathogenesis of FeChPV through further investigations and experimental infections. Furthermore, samples from cats with respiratory disease must be analyzed to discern the relationship between FeChPV infection and respiratory disease.

After analyzing the whole-genome sequences, the six strains identified in this study were found to be more closely related to other Chinese reference FeChPVs than to Canadian prototypes, thereby indicating that the predominant genotype of FeChPV strains are currently prevalent in China. The six obtained FeChPVs and other reference FeChPVs were classified as C*haphamaparvovirus Carnivoran2* after examining the evolutionary trees designed on the basis of NS1 and VP1 (14).

In a previous study, two FeChPVs were obtained from dogs suffering from diarrhea (2 of 285, 0.7%), suggesting that the virus has the ability of cross-host transmission (25). As reported in an Italian study, FeChPV (36.8%, 14/38) was the most frequently identified enteric virus, followed by FPV (23.7%, 9/38) (14). However, the prevalence rate of FeChPV in this study was lower than that reported in the aforementioned studies. This can be attributed to the difference in the years and regions from which the samples were collected. Additionally, 4 FeChPV-positive cats were coinfected with FPV, which was the predominant pathogen causing cat diarrhea in this study. Further epidemiological study is required to determine the pathogenic ability of FeChPV to cause diarrhea or other diseases in cats, because reports on FeChPV-induced diseases are scarce.

Notably, the recombination analysis performed in this study revealed that the two obtained FeChPV strains (CHN200228 and CHN180917, detected in cats in this study) were the close relatives to the recombinant parents of the CHN20201025 strain (detected in dogs). The clinical information of these strains present that all 6 FeChPV-positive cats in this study and two FeChPV-positive dogs in previous study were from different shelters. The high genome identities and recombinantion among the strains indicated that the virus might have spread to different species. Recombination studies suggest the genetic diversity of the FeChPV and supply crucial genetic information (37). Notably, the abundance of recombination events may denote that the mutation and recombination of FeChPV enhances its ability to adapt diverse hosts under natural selection conditions and the immune pressure in hosts resulting from prevalent and persistent infections.

When the tertiary structure models of CHN190305 and MN396757/IDEXX-1/CAN were compared, some discrepancies were predicted. For NS1 structural protein, Phe21Leu, Thr75Ala, and Lys267Glu might cause structural changes. Moreover, the tertiary structure alterations in VP1 for CHN1901011 and MN396757/IDEXX-1/CAN might occur due to His45Tyr, Ala57Ser, His419Asn/Thr, and Asp479Gly mutations. Importantly, NS1 is involved in viral replication, and VP1 is a capsid protein. The capsid affects the interaction between the virus and host receptors and plays a vital role in controlling the interactions (29). The aa mutation in one of the virus strains was identified in the conserved motif of NS1. Whether mutations in these sites affect FeChPV infectivity and cause changes in its function and pathophysiology needs further exploration.

In this study, the novel FeChPV was identified in 6 of 185 rectal swab samples and studied for understanding its circulation in Chinese cats with diarrhea. All identified strains belonged to the dominant cluster with FeChPV that was detected in China and other nations. These findings would aid in examining the prevalence of FeChPV and revealing the clinical symptoms may be caused by FeChPV.

## Data availability statement

The datasets presented in this study can be found in online repositories. The names of the repository/repositories and accession number(s) can be found in the article/Supplementary Material.

## Ethics statement

The animal studies were approved by Committee on the Ethics of Animal Experiments of South China Agricultural University (SYXK 2019-0136). The studies were conducted in accordance with the local legislation and institutional requirements. Written informed consent was obtained from the owners for the participation of their animals in this study.

## Author contributions

JJ and XX: conceptualization. HC: methodology and writing—original draft preparation. HC, KZ, and GG: software. XX, JJ, and LY: validation. JJ and KZ: formal analysis. ZZ and GG: investigation. QX and YB: resources. HC and ZZ: data curation and visualization. JJ and YK: writing—review and editing. HC and XX: supervision. LY and YK: project administration. LY: funding acquisition. All authors contributed to the article and approved the submitted version.

## Footnotes

^1^ http://jvenn.toulouse.inra.fr/app/example.html

^2^ https://www.chiplot.online/

^3^ https://wemol.wecomput.com/ui/#/

^4^ https://swissmodel.expasy.org/interactive
