# Supplementary material for: Molecular identification of carnivore chaphamaparvovirus 2 (feline chaphamaparvovirus) in cats with diarrhea from China
Source: Front Vet Sci. 2023 Oct 3;10:1252628. doi: 10.3389/fvets.2023.1252628 (PMC10580804; doi:10.3389/fvets.2023.1252628)
Supplement: Supplementary file 1 [file Table_1.DOCX]

Supplementary Material

**Molecular identification of Carnivore chaphamaparvovirus 2 (Feline chaphamaparvovirus) in cats with diarrhea from China**

**Hao Cui**^1,2^**, Zhibin Zhang**^1^**, Xin Xu**^1^**, Kejing Zuo**^3,*^**, Jun Ji**^1,*^**, Ge Guo**^1^**, Yunchao Kan**^1^**, Lunguang Yao**^1^**, Qingmei Xie**^4^**, Yingzuo Bi**^4^

**Correspondence:** [zuo_5502077@163.com；jijun020@126.com](mailto:zuo_5502077@163.com；jijun020@126.com)

# Supplementary Table 1. Primers used in this study for the detection or sequencing of viruses

| Primer name | Primer sequence (5′–3′) | Target | Location | Reference |
| --- | --- | --- | --- | --- |
| FeChPV-F1 | ATGTGGTTGTGTAGGACA | Sequencing | 36-53^a^ | This study |
| FeChPV-R1 | TCTCTTTCTAAACTGCGTCT |  | 940-959 |  |
| FeChPV-F2 | AACCTCTTCCATACGCAAA |  | 806-824 |  |
| FeChPV-R2 | CGCTGTCATCAATAGTCTCG |  | 1972-1953 |  |
| FeChPV-F3 | CTAATAAGCCCTGAATTAGCC |  | 1675-1695 |  |
| FeChPV-R3 | GCTTATATCAGTTGCCCAT |  | 3097-3115 |  |
| FeChPV-F4 | GAGCACAAGATGATGTCTATGA |  | 2904-2925 |  |
| FeChPV-R4 | TATGGGAGGGGGAATTGAAGTA |  | 4105-4126 |  |
| FeAstV-L1 | GGAGGTGGCTAAGGAGATAGT | Detection | Cap gene | (1) |
| FeAstV-R1 | CCTCTCTGAAGACGCCATGACT |  |  |  |
| FeAstV-L2 | CCCTCGAAGCGCTGGCACAA |  |  |  |
| FeAstV-R2 | CACCGAGCCCACCCCAGCTA |  |  |  |
| FBD1-L1 | TGACTCGTCTGTGGCGGGCT | Detection | VP1 gene | (1) |
| FBD1-R1 | TCGTTCGTGAGACGCTGCCA |  |  |  |
| FBD1-L2 | CAAAGGATCGGGAGCGGGCG |  |  |  |
| FBD1-R2 | TGCCCATGGTGTTGTGATTCCTATCCA |  |  |  |
| FeKoV-F | TGGAYTACAAGRTGTTTTGATGC | Detection | RdRp gene | (2) |
| FeKoV-R | ATGTTGTTRATGATGGTGTTGA |  |  |  |
| FPV-F | CAGGAAGATATCCAGAAGGA | Detection | Cap gene | (3) |
| FPV-R | GGTGCTAGTTGATATGTAATAAACA |  |  |  |
| CachaV-OF | CAACTAGCCGAATGCAGGGA | Detection | NS1 gene | (4) |
| CachaV-OR | CGATAACATCCCCGGACTGG |  |  |  |
| CachaV-IF | AGCTCAGTTTGGCCCAGATC |  |  |  |
| CachaV-IR | AGAGGGATCGCTGGATCTGT |  |  |  |
| FeChPV-OF1 | GGTGCGACGACGGAAGATAT | Detection | NS1 gene | (5) |
| FeChPV-OR1 | CAACACCACCATCTCCTGCT |  |  |  |
| FeChPV-OF2 | GCTGCAGTTCAGGTAGCTCA |  |  |  |
| FeChPV-OR2 | CAACACCACCATCTCCTGCT |  |  |  |

**^a^** The primer position is based on the sequence of the VRI-849 strain, GenBank accession number: MN794869.

# Supplementary Table 2. Prediction of recombination events of FeChPV strains in this study

| Recombination | Breakpoint | | Minor parent | | Major parent | | *P* |
| --- | --- | --- | --- | --- | --- | --- | --- |
|  | Start | End | Strains | Similarity | Strains | Similarity |  |
| CHN190305 | 1407 | 2089 | CHN191011 | 99.3% | MT708231/HF2/CHN | 99.2% | 5.59E^-05^ |
| CHN200523 | 1342 | 2221 | OP499832/C8-2/CHN | 99.3% | MZ031966/04/CHN | 99.6% | 3.27E^-04^ |
| MW404251/313R/ITA | 818 | 1533 | MT708231/HF2/CHN | 99.6% | CHN180917 | 98.7% | 9.02E^-05^ |
| CHN20201025 | 113 | 3823 | CHN200228 | 98.5% | CHN180917 | 99.5% | 3.12E^-04^ |

# Supplementary Table 3. Representative aa mutations in the NS1 of FeChPVs (“CHN”; this study) and the reference strain

| Strains | Substitution of aa mutations in NS1 | | | | | | | | | | |
| --- | --- | --- | --- | --- | --- | --- | --- | --- | --- | --- | --- |
|  | 21 | 35 | 75 | 121 | 250 | 267 | 271 | 372 | 411 | 506 | 580 |
| CHN180917 | F | M | T | H | T | K | N | T | R | N | N |
| CHN190305 | L | M | A | H | T | E | N | M | R | D | D |
| CHN191011 | F | T | T | H | T | E | N | M | I | N | N |
| CHN200228 | F | M | T | D | S | R | D | M | R | D | N |
| CHN200523 | F | M | A | H | S | K | D | M | R | D | N |
| CHN201109 | F | M | T | D | T | K | N | M | R | D | D |
| MN396757/IDEXX-1/CAN | F | M | T | H | T | K | N | M | R | D | D |
| OQ162042/CHN20201025/CHN | F | M | A | H | S | K | D | M | R | D | D |
| OQ162043/CHN20201226/CHN | F | M | A | H | T | K | N | M | R | D | D |
| MZ031966/04/CHN | F | M | T | H | T | K | N | M | R | D | D |
| MW404252/284R/ITA | F | M | T | H | T | K | N | M | R | D | D |
| MW404251/313R/ITA | F | M | T | H | S | K | N | M | R | D | D |
| MW404253/49E/ITA | F | M | T | H | S | K | N | M | R | D | D |
| MZ031965/AH-03/CHN | F | M | T | H | T | K | N | M | R | D | N |
| MT708231/HF2/CHN | F | M | T | H | S | K | N | M | R | D | D |
| OP499830/C7/CHN | F | M | A | H | S | K | D | M | R | N | D |
| OP499831/C8-1/CHN | F | M | A | H | S | K | D | M | R | N | N |
| OP499832/C8-2/CHN | F | M | A | H | S | K | D | M | R | N | N |
| OP499833/CBB/CHN | F | M | A | H | S | K | D | M | R | N | D |
| MN396757/IDEXX-1/CAN | F | M | T | H | T | K | N | M | R | D | D |
| MN794869/VRI849/USA | F | M | T | H | T | K | N | M | R | D | D |

# Supplementary Table 4. Representative aa mutations in the VP1 of FeChPVs (“CHN”; this study) and the reference strain

| Strains | Substitution of aa mutations in VP1 | | | | | | | | | | |
| --- | --- | --- | --- | --- | --- | --- | --- | --- | --- | --- | --- |
|  | 15 | 45 | 57 | 112 | 208 | 258 | 419 | 444 | 479 | 503 | 507 |
| CHN180917 | Y | Y | S | G | N | D | N | A | D | I | E |
| CHN190305 | C | H | A | R | N | D | N | A | D | I | E |
| CHN191011 | Y | Y | S | G | N | N | N | A | G | I | E |
| CHN200228 | Y | Y | A | G | D | D | T | A | D | N | D |
| CHN200523 | C | H | A | G | N | D | N | T | D | N | D |
| CHN201109 | C | H | A | G | D | D | N | A | D | N | D |
| MN396757/IDEXX-1/CAN | C | H | A | G | D | D | H | T | D | I | D |
| OQ162042/CHN20201025/CHN | C | H | A | R | N | D | H | A | D | A | D |
| OQ162043/CHN20201226/CHN | C | H | A | R | N | D | H | A | D | A | D |
| MZ031966/04/CHN | C | H | A | G | D | D | H | T | D | T | D |
| MW404252/284R/ITA | C | H | A | G | D | D | H | T | D | T | D |
| MW404251/313R/ITA | C | H | A | G | D | D | H | T | D | T | D |
| MW404253/49E/ITA | Y | H | A | G | D | D | H | T | D | T | D |
| MZ031965/AH-03/CHN | Y | Y | S | G | N | D | N | A | D | A | D |
| MT708231/HF2/CHN | C | H | A | G | D | D | H | T | D | T | D |
| OP499830/C7/CHN | C | Y | S | G | N | D | N | A | D | A | D |
| OP499831/C8-1/CHN | Y | Y | S | G | N | D | N | A | D | A | D |
| OP499832/C8-2/CHN | Y | Y | S | G | N | D | N | A | D | A | D |
| OP499833/CBB/CHN | Y | Y | S | G | N | D | N | A | D | A | D |
| MN396757/IDEXX-1/CAN | C | H | A | G | D | D | H | T | D | T | D |
| MN794869/VRI849/USA | C | H | A | G | D | D | H | T | D | T | D |

**REFERENCES**

1. Zhang W, Li L, Deng X, Kapusinszky B, Pesavento PA, Delwart E. Faecal virome of cats in an animal shelter. *J Gen Virol*. (2014) 95:2553-64. doi: 10.1099/vir.0.069674-0

2. Lu G, Zhang X, Luo J, Sun Y, Xu H, Huang J, et al. First report and genetic characterization of feline kobuvirus in diarrhoeic cats in China. *Transbound Emerg Dis*. (2018) 65:1357-63. doi: 10.1111/tbed.12916

3. Van Brussel K, Wang X, Shi M, Carrai M, Feng S, Li J, et al. The enteric virome of cats with feline panleukopenia differs in abundance and diversity from healthy cats. *Transbound Emerg Dis*. (2022) 69:e2952-2952e2966. doi: 10.1111/tbed.14646

4. Fahsbender E, Altan E, Seguin MA, Young P, Estrada M, Leutenegger C, et al. Chapparvovirus DNA Found in 4% of Dogs with Diarrhea. *Viruses*. (2019) 11. doi: 10.3390/v11050398

5. Li Y, Gordon E, Idle A, Altan E, Seguin MA, Estrada M, et al. Virome of a Feline Outbreak of Diarrhea and Vomiting Includes Bocaviruses and a Novel Chapparvovirus. *Viruses*. (2020) 12. doi: 10.3390/v12050506
