# Supplementary figures and images for: Molecular identification of carnivore chaphamaparvovirus 2 (feline chaphamaparvovirus) in cats with diarrhea from China
Source: Front Vet Sci. 2023 Oct 3;10:1252628. doi: 10.3389/fvets.2023.1252628 (PMC10580804; doi:10.3389/fvets.2023.1252628)

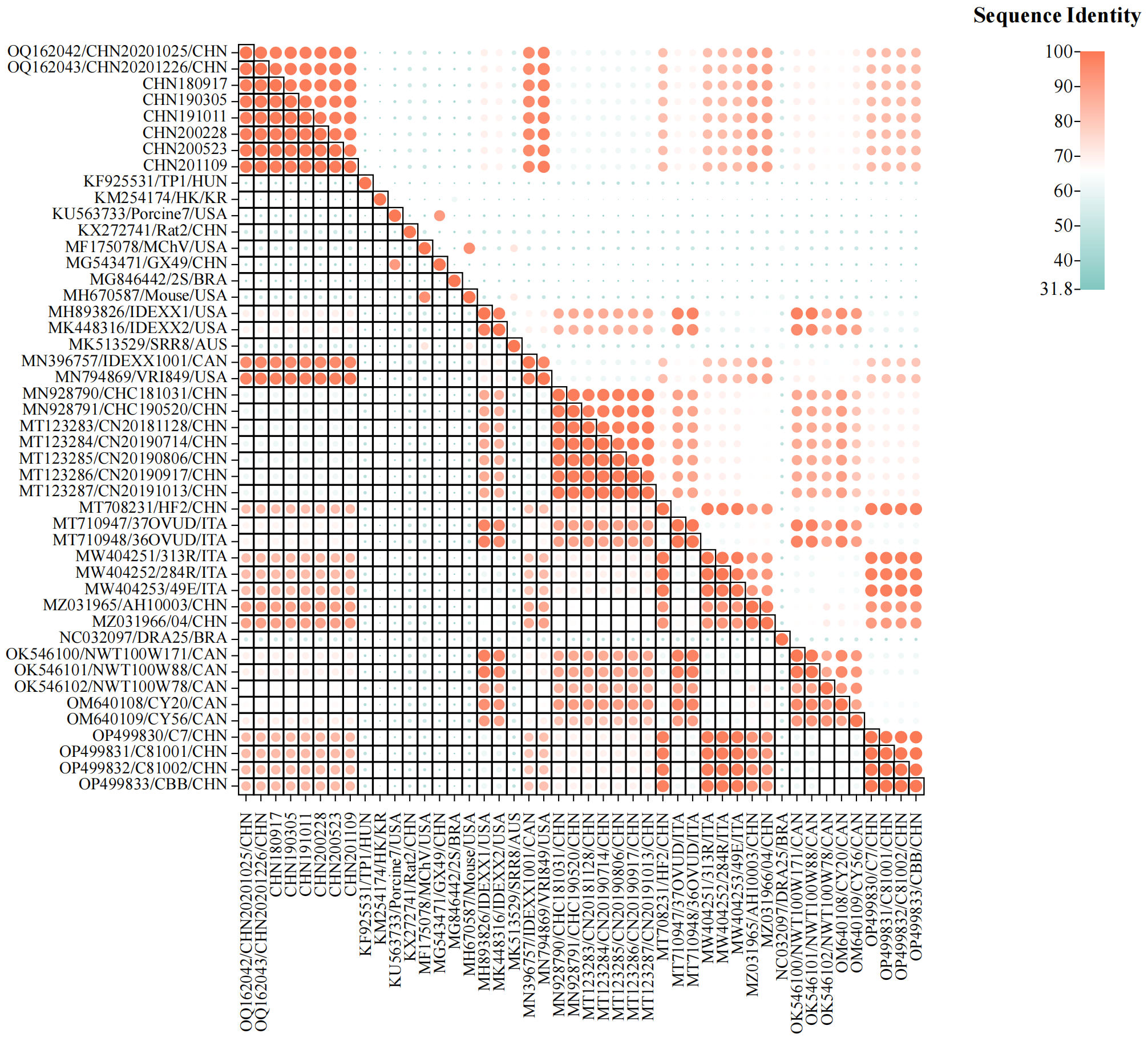

Supplement: Supplementary file 2 [file Image_1.TIF]
